# Lightweight consortium blockchain-enabled secured Vehicular ad Hoc Network using certificateless conditional privacy-preserving authentication mechanism

**DOI:** 10.1371/journal.pone.0310267

**Published:** 2024-10-31

**Authors:** Iqra Ilyas, Irfan ud Din, Abdullah Alourani, M. Usman Ashraf

**Affiliations:** 1 Department of Computer Science, Superior University, Lahore, Pakistan; 2 Department of Computer Science, New Uzbekistan University, Tashkent, Uzbekistan; 3 Department of Management Information Systems, College of Business and Economics, Qassim University, Buraydah, Saudi Arabia; 4 Department of Computer Science, GC Women University Sialkot, Sialkot, Pakistan; Cardiff Metropolitan University, UNITED KINGDOM OF GREAT BRITAIN AND NORTHERN IRELAND

## Abstract

Towards the intelligent transportation systems, Location Based Service (LBS) are widely engaged in Vehicular Ad Hoc Networks (VANETs) that are becoming as significant application that change the human driving experience in today’s world. LBS systems facilitate the users with intelligent services by collecting an accurate location information. Due to the frequent exchange rate of the location information in an open environment, VANETs are inherently susceptible to privacy and security attacks. In past, many schemes have been proposed to ensure the privacy and security of exchanged location information; but fail to deploy in practical VANETs. At the same time, system efficiency is compromised which is another primary requirement of VANETs. Leveraging the semi-decentralized and lightweight nature of consortium blockchain technology, and Certificateless conditional privacy protection scheme to reduce the node authentication overhead, this paper introduces **C**onsortium **B**lockchain assisted **C**ertificateless **C**onditional **P**rivacy **P**rotection scheme to address the aforementioned challenges. Additionally, the proposed scheme has ability to develop anonymous regions for a particular time stamp ensuring the location privacy of vehicles. Rigorous security analysis and experiments show the practicality and resilience to various attack models, and achieve ADP 83% with maximum malicious attacks. Comparing with existing state of the art methods, the proposed scheme exhibits the privacy improvement and low computational complexity.

## I. Introduction

The rapid development of Vehicular Ad Hoc Networks (VANETs) has revolutionized modern transportation by enabling vehicles to communicate with each other and with roadside infrastructure. This communication facilitates a wide range of applications, from traffic management and safety services to infotainment systems. VANETs significantly enhance advanced traffic systems by enabling real-time traffic management, improving road safety, reducing environmental impact, and enhancing the driving experience. They facilitate dynamic communication among vehicles and infrastructure, optimizing traffic flow and providing timely safety warnings [1, 2].

**Fig 1:**
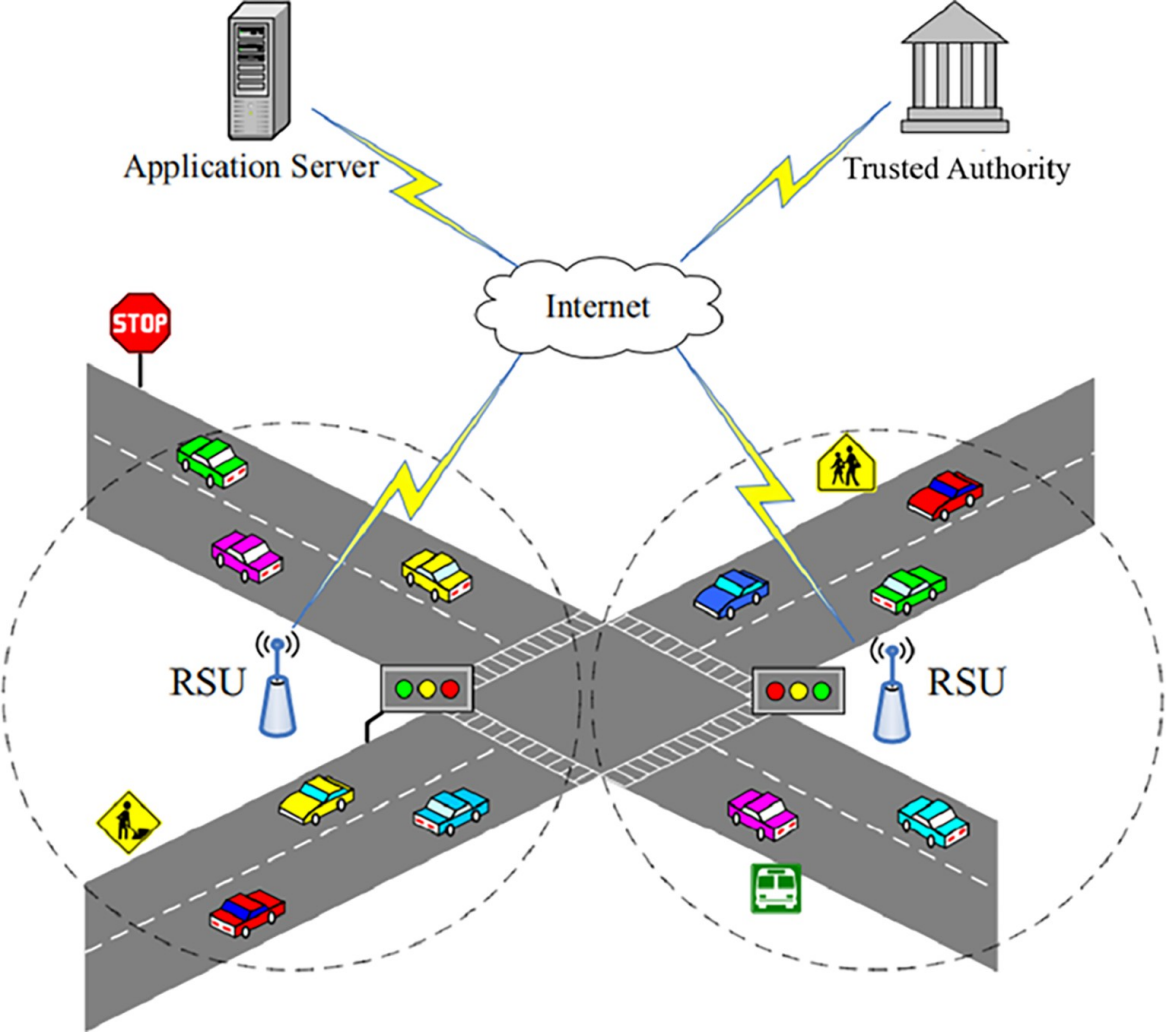
A fundamental structure of VANETs.

According to a fundamental architecture of VANET system shown in Fig 1, a VANET system comprising roadside units (RSUs), vehicles equipped with On-Board Units (OBUs), a trusted authority (TA), and an applications server such as a traffic control center, communication and coordination are facilitated through a multi-layered structure. At the lower layer, RSUs and OBUs form the backbone of the network, enabling direct communication between vehicles and with roadside infrastructure. RSUs serve as communication hubs, relaying data between vehicles and the top layer infrastructure. OBUs installed in vehicles allow for seamless integration into the VANET, enabling real-time data exchange and collaboration. At the top layer, the trusted authority oversees security and authentication processes, ensuring the integrity of communications and protecting against unauthorized access. Meanwhile, the applications server hosts a variety of services and applications that utilize data from the VANET, such as traffic management, emergency assistance, and navigation systems. Communications within the VANET primarily involve two modes: vehicle-to-vehicle (V2V) and vehicle-to-infrastructure (V2I). V2V communication enables direct interaction between vehicles, facilitating cooperative driving, collision avoidance, and platooning. V2I communication, on the other hand, involves communication between vehicles and roadside infrastructure, supporting services such as real-time traffic updates, road condition monitoring, and remote vehicle diagnostics. Together, these components and communication modes form a comprehensive VANET system aimed at improving traffic efficiency, enhancing road safety, and providing a seamless driving experience [3].

Location-Based Services (LBS) play a crucial role within VANETs by offering real-time navigation [4], context-aware safety alerts [5, 6], geographic messaging, resource management, personalized services, and efficient emergency response [7–9]. Together, VANETs and LBS form the backbone of intelligent transportation systems, leading to safer, more efficient, and environmentally friendly road networks. On other hand, VANETs are inherently susceptible to privacy and security attacks when a vehicle’s accurate location information is disclosed. For instance, a vehicle A is driving on a busy highway during rush hour. Vehicle A’s LBS system provides real-time traffic updates and suggests an alternate route to avoid congestion ahead. However, to calculate the optimal route, Vehicle A’s current location must be disclosed to the LBS system. In this scenario, Vehicle A’s location is disclosed to the LBS system for the purpose of receiving navigational assistance. The LBS system analyzes Vehicle A’s location along with traffic data from other vehicles in the area to determine the most efficient route. While this disclosure of location is necessary for providing accurate and timely navigation services, it raises privacy concerns as Vehicle A’s location is shared with the LBS provider. A vehicle user’s privacy is much concerned with driving route. Upon keeping the route as secret, serious consequences may be occurred by linking multiple broadcasted messages. Therefore, ensuring secure communication, modern VANET systems often incorporate conditional privacy-preserving (CPP) mechanisms [10–14].

Usually on road network, due to less computation power and storage capacity of OBUs, thousands of the request messages are sent over RSUs in every second. In this scenario, certificate generating mechanism affect the overall efficiency in the system, and batch verification is preferred using certificateless aggregate signature (CLAS) approach. Using CLAS, a variety of schemes have been proposed in recent years [15–18]. Though these schemes improve the privacy and security with some extent of efficiency in VANETs; but they all are designed with unique authentication node that cause the loss of authentication effectiveness once a vehicle enters into a new region. To overcome this issue, researchers propose various schemes using cross-authentication algorithm [19]. These state-of-the-art methods inherently face interaction challenges among the authentication nodes, OBUs, and RSUs. Nevertheless, a trust relationship among the authentication nodes is required to be developed by some trusted third parties. In this context, blockchain technology offers a robust and decentralized framework that can be the best solution for cross-authentication. Thus, a variety of blockchain based cross authentication schemes are introduced [20–24]. Generally, blockchain is categorized into three types such as Public blockchain (PBC), private blockchain, and lightweight consortium blockchain. Comparing the Public and consortium blockchains, public blockchains (PBC), like Bitcoin and Ethereum, are open to anyone and operate in a decentralized manner, where anyone can participate in the consensus process. They are highly transparent and secure due to their broad participant base but often suffer from slower transaction speeds and higher energy consumption. On the other hand, consortium blockchains (CBC) are semi-decentralized, where a pre-selected group of nodes from multiple organizations manages the consensus process. This type of blockchain is not open to everyone, ensuring higher efficiency and faster transaction speeds while maintaining a degree of decentralization. Consortium blockchain is particularly advantageous when consensus rules are compulsory to be considered, as they provide a controlled environment that balances transparency and performance. Therefore, leveraging the semi-decentralized and lightweight nature, we use lightweight consortium blockchain technology in this study, and propose lightweight consortium blockchain and CLAS based CPP scheme to address the critical issues of security, privacy, and efficiency in VANETs. Extensive security analysis and performance evaluations demonstrate that the proposed scheme provides robust protection against various security threats, including impersonation, replay, and Sybil attacks, while maintaining low computational and communication overhead. The results indicate that our proposed scheme not only enhances the security and privacy of VANET communications but also improves the overall efficiency and reliability of the network.

### Our contribution

To address the above privacy and efficiency related challenges, this paper introduces a balanced construction of consortium blockchain with CLAS-CPP scheme. Further we summarize the key contribution as follows:

1. While preserving vehicles privacy in VANETs, realizing unlinkability. Instead of using real identity, we adopt pseudonym identity mechanism. In results, an adversary is unable to track the actual identity of a vehicle.
2. leveraging the semi-decentralized and lightweight nature of consortium blockchain (CBC) where a pre-selected group of nodes from multiple organizations manages the consensus process and much feasible for cross authentication, we couple the RSUs using consortium blockchain technology ensuring higher efficiency and faster transaction speeds while maintaining a degree of decentralization.
3. Traceability is another primary factor to be performed when illegal activities are found in VANET system. For this purpose, we adopted trusted tracing authority (TA) that keeps the record of all registered vehicles and can trace easily in case of abnormal situation.
4. Widespread adoption of certificate based signature scheme affects the system efficiency. Therefore, we use certificateless aggregate signature (CLAS) along with conditional privacy protecting (CPP) approach that increase the system efficiency as well as privacy.
5. Achieved higher privacy and efficiency as compared to existing state of the art schemes. We conduct both theoretical analyses and simulation experiments in real road networks environment using OMNET++ simulation tool.

### Organization

Rest of the paper is organized in such way that section II describes the existing privacy and security related challenges for VANETs considered in this paper. Section IV presents the preliminaries for this research. Further, section V demonstrate the proposed scheme comprehensively. Section VI describe a rigorous security analysis of our proposed scheme. Section VIII present an extensive experiments and results. In section IX, we conclude the research with future directions and opportunities.

## II. Challenges for VANETs

An open nature of VANETs interacting with LBS systems, coupled with the mobility of nodes, faces variety of security and privacy challenges such as.

### Query content modification

In VANETs, the query content modification issue arises when the data transmitted between vehicles or between vehicles and infrastructure nodes is altered, either maliciously or unintentionally. For instance, the query passes through multiple relay nodes before reaching the server. An attacker, who has compromised one of these relay nodes, intercepts and modifies the content of the query. Instead of requesting the fastest route, the modified query asks for an alternative, less efficient route. The server, unaware of the modification, processes the query and sends back directions based on the false request. As a result, the user’s vehicle follows a suboptimal route, potentially leading to increased travel time and fuel consumption. This problem can compromise the reliability and integrity of the information shared across the network, such as traffic conditions, safety alerts, and navigation instructions. Malicious nodes might inject false data to create confusion, cause accidents, or manipulate traffic flow for personal gain, while accidental modifications can result from signal interference or transmission errors. To address this, robust security protocols and data validation mechanisms are essential to ensure the authenticity and accuracy of the exchanged information, thereby maintaining the overall safety and efficiency of the VANET system [25].

### Content replay

Content replay refers to the malicious retransmission of previously captured valid messages. This form of attack severely disrupts the network’s functionality, as outdated or irrelevant information is injected back into the system, causing confusion and potentially hazardous situations. For example, a replayed emergency braking signal could trigger unnecessary braking actions by other vehicles, leading to accidents or traffic congestion. The effectiveness of the VANET system relies on the timeliness and accuracy of data, so replay attacks undermine these core principles. To mitigate this risk, VANETs must implement strong authentication and time-stamping mechanisms, ensuring that each message is both from a legitimate source and temporally relevant, thereby protecting the network against replay attacks and maintaining its integrity and reliability [26].

### Impersonation

Impersonation issue arises when an attacker masquerades as a legitimate vehicle or network node, gaining unauthorized access to the network. This can lead to severe security breaches, as the impersonator can inject false information, manipulate traffic patterns, or even disable critical safety messages. For instance, an attacker might pose as an emergency vehicle to force other cars to yield or change lanes, creating chaos and potentially causing accidents. Impersonation compromises the trust and reliability that VANETs depend on to function effectively. To counteract this, VANETs require robust identity verification protocols and secure cryptographic methods to authenticate the identity of each node, ensuring that all participants in the network are genuine and authorized, thus protecting the system from such malicious exploits [27].

### Efficiency

The efficiency issue in Vehicular Ad Hoc Networks (VANETs) pertains to the network’s ability to handle high volumes of data traffic and deliver timely, reliable communication among vehicles and infrastructure nodes. Given the dynamic and highly mobile nature of VANETs, maintaining efficient data transmission is crucial for real-time applications like collision avoidance, traffic management, and navigation assistance. Challenges include managing the rapidly changing network topology, mitigating packet loss and delays, and optimizing bandwidth usage to prevent congestion. Efficient routing protocols, adaptive communication strategies, and robust data dissemination methods are essential to address these challenges. Ensuring high efficiency in VANETs not only enhances the overall user experience but also significantly contributes to road safety and traffic flow optimization, making the network resilient and effective in diverse and demanding conditions [28].

Note that privacy challenges for VANETs are not limited to aforementioned but also include some substantial issues such as data confidentiality, data availability etc. However, these challenges are beyond the scope of this research.

## III. Related work

To satisfy two primary attributes such as privacy and efficiency in VANETs, variety of techniques have been proposed in recent past. Since last decade, protecting privacy becomes a critical necessity when dealing with sensitive traffic-related messages, demanding measures against misuse or unauthorized disclosure in VANETs. In the domain of vehicular communication, the examination of privacy issues spans various interaction levels, covering aggregation, processing, collection, evaluation, and visualization. Given the sensitivity of the exchanged information, the preservation of privacy emerges as an imperative requirement.

Noteworthy inquiries in this field involve Ming and Shen [29] certificateless conditional privacy protection scheme based on elliptic curve. The certificateless conditional privacy protection scheme based on elliptic curve cryptography offers advantages such as eliminating certificate management overhead, enhancing privacy through conditional access control, and reducing vulnerability to key compromise. Leveraging elliptic curve cryptography strengthens security while simplifying key management. However, challenges include the complexity of implementation, limited interoperability with traditional PKI systems, the risk of misconfiguration leading to security vulnerabilities, and dependency on specific cryptographic algorithms, which may impact long-term security and compatibility. However, this scheme falls short of meeting maximum privacy requirements. Shawky, Mahmoud A., et al. [30] cross layer authentication based privacy provisioning scheme prioritizes vehicle revocation over certificate revocation but overlooks contextual privacy. Authors introduced low-complexity cross-layer authentication scheme for VANET applications, leveraging short-term channel reciprocity and randomness for reauthentication to mitigate performance limitations, especially the substantial overhead of signature generation and verification. In [31], Liu, et al. introduced PTAP (privacy-preserving traceable authentication protocol). The PTAP addresses weaknesses and offers additional benefits. It employs semi-honest entities to facilitate stakeholder interaction without needing a trusted third party, resulting in very low computational costs. Additionally, the PTAP ensures vehicles can access remote services while maintaining identity anonymity and location privacy. However, these schemes may also introduce challenges. For instance, the anonymity provided may hinder certain location-based services or functionalities that rely on accurate location information. Moreover, ensuring the effectiveness of such schemes while maintaining usability and efficiency can be complex, requiring careful design and implementation to balance privacy concerns with practicality and functionality. Therefore, the proposed scheme disregards unlinkability and observability. Although Manickam et al. [32] FC-CPPA (Fog Server based conditional privacy protecting authentication) scheme ensures communication efficiency and vehicle privacy, it lacks contextual privacy. Zhou et al., [33] Safety-related Privacy Scheme (SRPS) focus on group communication without fully addressing contextual requirements. Similarly, Dongxu Zhu and Yepeng Guan [34] proposed a lightweight conditional privacy-preserving identity authentication scheme (LCPIA) that achieve anonymity but falls short on unlinkability and unobservability. Alazzawi et al.’s [35] proposed pseudo identity privacy preservation method. One advantage of pseudo identity mechanism is that these schemes enable data analysis to be conducted without directly exposing individuals’ sensitive information, thus preserving their privacy. By replacing real identities with pseudo-identifiers, such as randomly generated numbers or codes, individuals can participate in data analysis without revealing personal details. This approach can help mitigate concerns about data breaches or unauthorized access to sensitive information. However, a key disadvantage is the potential loss of data accuracy, utility, and contextual privacy. Similarly, Bayat et al.’s [36] RSU-based scheme assumes drivers’ preference for anonymity but neglects unobservability.

Wang, Zhihua, et al., [37] devised a novel data sharing scheme for 6G-VANET. The design encompasses a VANET architecture that integrates 6G, SDN, edge computing, and other technologies. The effective deduplication of shared data is achieved through the application of word2vec. Two distinct blockchain forms are introduced to store essential information throughout the data sharing process. The vehicle layer, functioning as the SDN data plane, is responsible for aggregating diverse shared data. The SDN control plane, deployed with MEC, focuses on calculating vehicle set matching and blockchain block production operations. Leveraging MEC’s computational storage resources, word2vec, and two blockchains are embedded, harnessing the power of edge intelligence. To reflect the decentralized nature of blockchain and alleviate pressure on the SDN control plane, a distributed design for the SDN control plane is implemented based on geographical regions, deploying the certification center and other service co-providers in the SDN application plane. In future endeavors, the proposed solution will undergo performance evaluations on alternative simulation platforms. Additional considerations for data security protection, such as state access, will be explored, along with expanding application services on the blockchain platform. Moreover, the integration of the data sharing mechanism and reputation value will be explored to create a more secure and efficient data sharing system.

Ahmed, W., Di, W. and Mukathe, D., [38] presented a robust authentication scheme for anonymous message transmission in vehicular networks, ensuring privacy protection. The system allows the Trusted Authority (TA) to trace and eliminate anonymous malicious vehicles. A trust management model empowers the RSU to verify vehicle node and traffic data credibility, identifying malicious vehicles and selectively broadcasting genuine events. The RSU calculates the sender’s trust value, distinguishing malicious from honest senders based on trust values and anomaly ratios. Trust values are recorded in a blockchain, representing RSU consensus on each vehicle’s trustworthiness. Reports on malicious vehicles are communicated to the TA. The system excels in accurately evaluating data and vehicle trustworthiness, identifying malicious senders, and ensuring the broadcast of genuine events in VANETs. However, addressing the ’node initialization’ challenge in trust management is still an open challenge for future endeavors where every new node joining the network needs an initial trust value assigned. Existing trust management solutions frequently rely on assuming a static initial value for trust when encountering a new node. Assigning initial trust values to new nodes within a vehicular network poses a substantial and intricate challenge in trust management models.

Shareeda, et al., [39] employing the Chinese remainder theorem proposed a VANET security system that ensures the security of transmitted messages by dynamically and adaptively responding to vehicles and RSUs entering the VANET. It presented a solution for forensics and tracing of accident vehicles based on smart contracts. Additionally, the scheme undergoes thorough security proof and analysis to verify its adherence to VANET’s security requirements. However, the proposed scheme still faces several challenges of privacy while interacting with the system for any point of interest.

Below Table 1 presents a comparative analysis of existing privacy provisioning schemes discussed in related work section.

**Table 1:** Comparative analysis of existing methods toward privacy in VANET systems.

Ref.	Proposed Solution	Strength	Weaknesses	Type of Model	BC
[29]	CLS-MR scheme	less certificate management overhead	Used bilinear pairing	Entity-centric	No
[30]	Cross-Layer	Efficient with Low delay.	Data availability not determined	Hybrid	No
[31]	PTAP	Improved VANET performance	No resistance type II attacks	Hybrid	Yes
[32]	FC-CPPA	Reduce certificate management overhead.	Unevaluated data.	Hybrid	Yes
[33]	SRPS	Resist type II attacks	Data availability not determined	Entity-centric	No
[34]	LCPIA	Improved performance and privacy.	Fail to consider security	Entity-centric	No
[35]	pseudo-identity based scheme	Traditional Authentication	Both privacy and efficiency issues.	Hybrid	No
[36]	NERA	Without bilinear pairing	Certificate management overhead.	Entity-centric	No
[37]	6G-VANET	Lightweight message authentication	No real implementation.	Entity-centric	Yes
[38]	blockchain-based authentication	Identify privacy integrity	Low performance.	Entity-centric	Yes
[39]	CRT based authentication	Improved privacy authentication.	Unevaluated Data	Entity-centric	Yes

Recognizing shortcomings in existing schemes, we propose a generic consortium Blockchain assisted Certificateless Conditional Privacy Protection scheme for VANETs to address the contextual privacy requirements, encompassing anonymity, unlinkability, and unobservability. By leveraging Leveraging the decentralized and immutable nature of blockchain technology, and Certificateless conditional privacy protection scheme to reduce the node authentication overhead, our scheme withstands various attacks, ensuring a robust solution that satisfies both privacy and efficiency in VANETs.

## IV. Preliminaries

In the subsequent sections, we introduce the essential mathematical tools employed in this investigation. Subsequently, we delve into the model governing vehicular communication and explore the adversary models. Finally, we articulate the security and privacy requirements integral to the proposed scheme.

### A. Certificateless Aggregate Signature (CLAS)

Certificateless Aggregate Signature (CLAS) significantly enhances privacy protection in Vehicular Ad Hoc Networks (VANETs) by eliminating the need for certificates while enabling efficient and secure communication [40]. In VANETs, vehicles *V*_*i*_​ communicate by sending signed messages *m*_*i*_​ to other entities such as Roadside Units (RSUs) and other vehicles *V*_*j*_​. Using a certificateless approach, each vehicle *V*_*i*_​ generates a partial private key *K*_*priv*_​ without relying on a certificate authority. This key is used to produce an individual signature *σ*_*i*_ for the message *m*_*i*_​. Multiple signatures *σ*_*i*_ ​ from different vehicles are then aggregated into a single compact signature *σ*_*agg*_ represented as $\sigma agg=\prod_{i=1}^{n}\sigma i$, where *n* is the number of individual signatures. The aggregated signature *σ*_*agg*_​ is verified using an aggregated public key *PK*_*agg*_​, ensuring that all *n* messages *m*_*i*_​ are authentic and untampered. This method reduces the communication overhead and computational cost associated with handling multiple certificates and signatures, thereby preserving vehicle anonymity and enhancing privacy. The elimination of certificates mitigates the risk of certificate-related attacks and simplifies key management, making CLAS an effective solution for maintaining security and privacy in VANET environments.

### B. Conditional privacy-preserving

In VANETs, Conditional Privacy-preserving (CPP) mechanisms are designed to balance privacy and traceability without relying on traditional Public Key Infrastructure (*PKI*) [41]. Let *V = {v*_*1*_,*v*_*2*_,*…*,*v*_*n*_*}* represent the set of vehicles, and P denote the trusted Private Key Generator (PKG). Each vehicle *v*_*i*_ is assigned a partial private key *di* by P and generates its own secret value *x*_*i*_ and public key *P*_*i*_ *= x*_*i*_*⋅G*, where *G* is a generator of the elliptic curve group. The vehicle’s full private key is *S*_*i*_ *= (d*_*i*_,*x*_*i*_*)*. For conditional privacy, each message *m* sent by vehicle *v*_*i*_ includes a pseudonym *P*_*i*,*t*_ ​ valid for a time period t, computed as *P*_*i*,*t*_ *= H(t)⋅P*_*i*_ where *H* is a hash function. If misbehavior is detected, an authority *A* can request the *PKG* to reveal *v*_*i*_*​’s* identity by combining *di* ​ and *Pi* ​ to trace the pseudonym *Pi*,*t* back to the vehicle. The CPP mechanism ensures privacy by keeping *x*_*i*_ secret while enabling accountability through the conditional traceability provided by P and A. This balance maintains security and privacy efficiently without certificates, enhancing trust in VANET communications.

### C. Lightweight consortium blockchain

In Vehicular Ad Hoc Networks (VANETs), maintaining the security and trustworthiness of data can be effectively achieved through a lightweight consortium blockchain framework. In blockchain emerging technology, the decentralized nature of the identity management system ensures that no single entity has undue control over the identity information, providing a secure and transparent framework for managing identities in a distributed manner [42–45]. Let *D* represent the set of participants in the blockchain-based decentralized identity management system, where *D* = {*d*_1_​,*d*_2_​,…,*d*_*n*_​}, and *n* is the total number of participants. Let *K* denote the set of cryptographic key pairs associated with each participant in the system, where *K* = {(*pk*_1_​,*sk*_1_​),(*pk*_2_​,*sk*_2_​),…,(*pk*_*n*_​,*sk*_*n*_​)}, and *pk*_*i*​_ and *sk*_*i*_​ represent the public and private keys of participant *di*​, respectively. The identity of each participant *di*​ is represented as *ID*_*i*_​, where *ID*_*i*​_ is a unique identifier associated with participant *di*​ in the blockchain. Transactions in the system are denoted by *T*, and each transaction *t* includes relevant information such as the sender’s identity *ID*_*sender*_​, receiver’s identity *ID*_*receiver*_​, and the content of the transaction. The blockchain is represented as *B*, and it consists of a sequence of blocks {*B*_1_​,*B*_2_​,…,*B*_*m*_​}, where each block *B*_*j*_​ contains a set of transactions{*t*_1_​,*t*_2_​,…,*t*_*k*_​} and a reference to the previous block *B*_*j*−1_. The consensus mechanism used for block validation and addition is denoted by *C*, and it ensures agreement among participants on the validity of transactions and the order of blocks in the blockchain. The smart contract functionality is denoted by *S*, and it defines the rules and conditions for executing transactions automatically based on predefined terms within the blockchain.

## V. Proposed method

This section elaborates the proposed Lightweight consortium Blockchain and Certificateless conditional privacy protecting scheme we put forth. Before discussing the proposed scheme, we first present the terms used in research demonstrated in Table 2.

**Table 2:** Nomenclature.

Notation	Description
*ID* _ *user* _	Identity of a user in the decentralized identity management system.
*IDRSU*	Identity of a Roadside Unit (RSU) in the decentralized identity management system.
*IDTA*	Identity of the Trusted Authority (TA) in the decentralized identity management system.
IDRSU*	Pseudonymous identity of an RSU in the decentralized identity management system.
*ID* _ user* _	Pseudonymous identity of a user in the decentralized identity management system.
*EC* _ *pub* _	Public key in elliptic curve cryptography for secure data transmission.
*EC* _ *priv* _	Private key in elliptic curve cryptography for secure data transmission.
*Consensus(T* _ *1* _ ,*T*_ *2* _ ,. *T*_ *n* _ *)*	Consensus function in the consensus mechanism for validating and authorizing transactions.
*T*	Set of all proposed transactions in the consensus mechanism.
*T* _ *i* _	Set of transactions proposed by participant *i* in the consensus.
*T* _ *agreed* _	Agreed set of transactions in the consensus.
*Validate (Tj)*	Validation function for transaction T*j* in the consensus mechanism.
*B*	Newly formed block in the consensus.
*CBC*	consortium blockchain
*LCBC*	lightweight consortium blockchain
*OBU*	Onboard Unit

**Fig 2:**
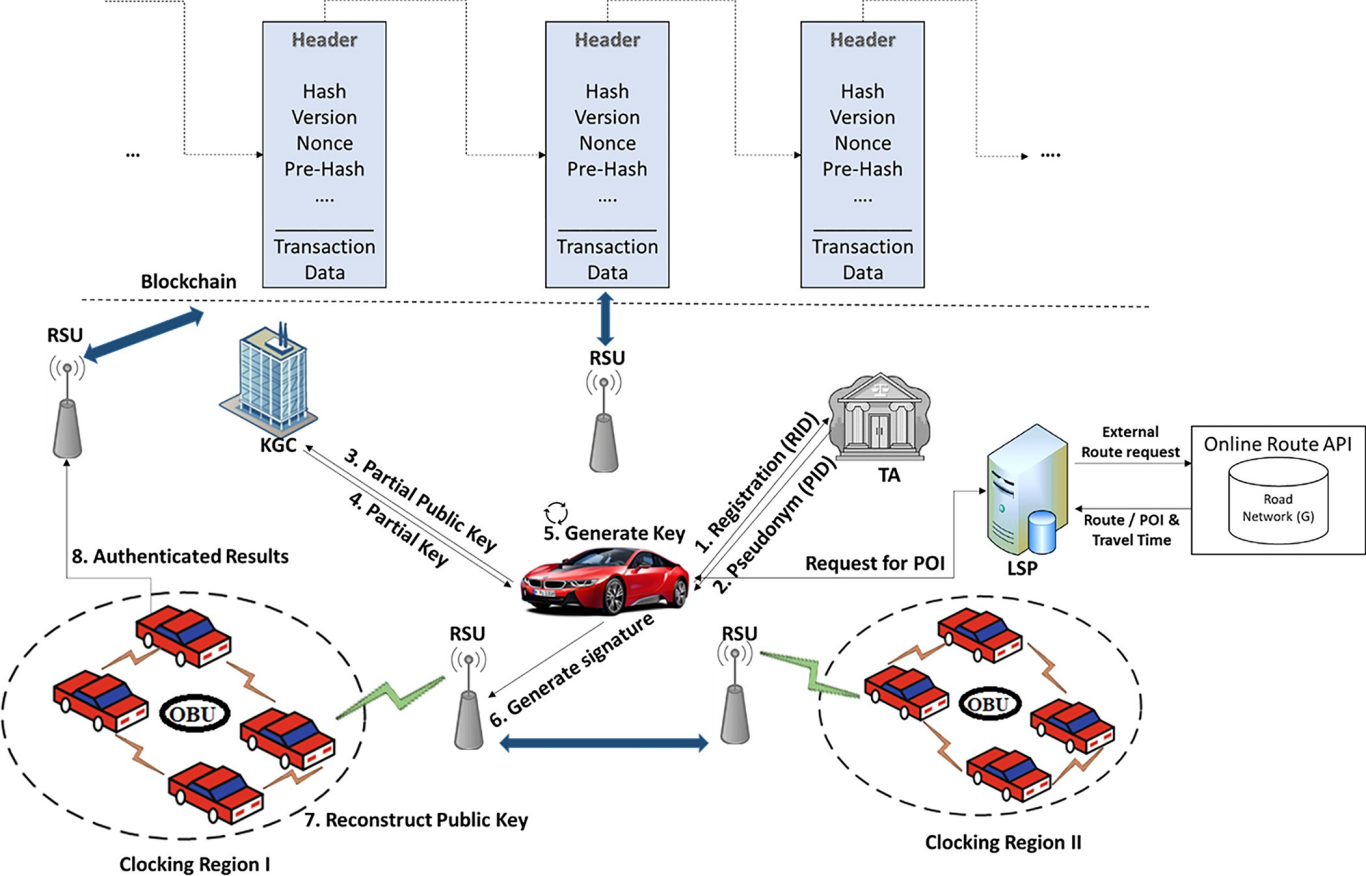
Block diagram of proposed scheme.

Conventionally, a VANET system consists of vehicles, Onboard Units (OBUs), Roadside Units (RSUs), Traceable authority (TA), and key generation center (KGC) that are connected each other through internet. Among these entities, TA and KGC are always assumed as trusted third parties. Usually, existing state-of-the-art methods adopt different types of privacy protecting techniques including identity (ID) [46–48], public key infrastructure (PKI) [49–52], and certificateless aggregate signature (CLAS) [53–55] based approaches in VANETs. Though each scheme has its own pros and cons. For instance, PKI affect the system efficiency while managing the resources and certificates. On other hand, ID base method also face the key escrow challenges. To overcome these vital challenges, this paper adopt CLAS based conditional privacy protection (CPP) mechanism that emerges as a potential solution. Although CLAS based CPP scheme improve the privacy and security with some extent of efficiency in VANETs; but they all are designed with unique authentication node that cause the loss of authentication effectiveness once a vehicle enters into a new region. To address this issue, we use lightweight consortium blockchains (CBC) which is not open to everyone, ensuring higher efficiency and faster transaction speeds while maintaining a degree of decentralization. Consortium blockchain is particularly advantageous when consensus rules are compulsory to be considered, as they provide a controlled environment that balances transparency and efficiency. Detail architecture of proposed scheme is described in Fig 2.

Further the detail of each entity is described as follows:

### Vehicles (V)

In Vehicular Ad Hoc Networks (VANETs), vehicles equipped with On-Board Units (OBUs) interact to enhance transportation efficiency and safety. OBUs enable vehicles to communicate with each other (Vehicle-to-Vehicle, V2V) and with roadside infrastructure (Vehicle-to-Infrastructure, V2I). This interaction facilitates the exchange of critical information such as traffic conditions, accident alerts, and navigation assistance in real time. By leveraging OBUs, VANET systems improve traffic flow, reduce the likelihood of accidents, and provide drivers with timely information, thereby creating a more connected and intelligent transportation network.

### Tracing Authority (TA)

TA plays a pivotal role in maintaining network integrity and security. The TA is responsible for overseeing and managing the network to ensure that the information disseminated is accurate and reliable. If a vehicle disseminates incorrect or malicious information, the TA can trace the source using cryptographic techniques and digital signatures embedded in the messages. By verifying these signatures against a database of registered vehicles, the TA can identify and locate the offending vehicle, thereby maintaining trust and accountability within the network. This capability is essential for preventing misinformation, ensuring the safety of all users, and upholding the effectiveness of VANET systems.

### Key Generation Center (KGC)

KGC is responsible for generating and distributing cryptographic keys that vehicles use to authenticate and encrypt their communications. To generate part of a vehicle’s private key, the KGC calls the Partial-Private-Key-Extract (*PPKE*) function, which involves creating a partial private key using specific cryptographic algorithms and parameters. This partial key is then securely transmitted back to the vehicle. The vehicle combines this partial key with its own unique identifier or other secret information to form its complete private key. This process ensures that even if the partial key is intercepted, it cannot be used alone to compromise the vehicle’s communications, thus enhancing the overall security of the VANET system.

### On-Board Units (OBUs)

OBUs play a crucial role in Vehicular Ad-Hoc Networks (VANETs) by enabling communication between vehicles and with roadside infrastructure. OBUs, installed in vehicles, facilitate the exchange of information such as traffic conditions, safety warnings, and navigation assistance. They collect data from various sensors within the vehicle and communicate this information to other vehicles or Roadside Units (RSUs). OBUs forward information to RSUs using Dedicated Short-Range Communications (DSRC) or other wireless communication protocols. This interaction allows for real-time updates on road conditions and enhances overall traffic management and safety by creating a connected network of smart vehicles and infrastructure.

### Consortium Blockchain (CBC) based Roadside Unit (RSU)

Suppose that RSUs are extensively deployed on road networks. When a vehicle moves into a different geographic area, it needs to establish trust and secure communication with the local infrastructure and other vehicles. Ensuring continuous access to vital data for vehicles and facilitating the swift recording of new trust information, CBC facilitates this by maintaining a distributed ledger shared among a consortium of trusted entities, such as local authorities and service providers. By integrating RSUs into the vehicular network, a blockchain core network emerges, storing multiple encoded blocks within the RSUs and forming a comprehensive, decentralized database. Even if a vehicle travels out of the initial RSU’s communication range due to high-speed movement, it can still communicate with other RSUs to retrieve query results. RSUs manage query requests from vehicles, creating anonymous cloaking regions and forwarding them to the Location Service Provider. Additionally, RSUs return the query results to the requesting vehicle(s) and are responsible for maintaining the blockchain.

Considering the features of above discussed entities, the operational sequence of proposed scheme is summarized more specifically as follows:

1. In first step, overall the system is initialized, and relevant parameters are disseminated among all connected entities which is the requirement for all vehicles to get registration with *TA*. Vehicle (*V*_*i*_*)* requests to *TA* for registration along with its real identity (*RID*_*i*_*)*.
2. *TA* executes the pre-configured pseudonym generation algorithm, and generate a pseudonym identity (*PID*_*i*_*)* for the requesting entity *V*_*i*_. Further *V*_*i*_ preloads the *PID*_*i*_ over *OBU*.
3. Using secret-value algorithm, *V*_*i*_ generates private key *(K*_*priv*_*)* for itself. Further, *V*_*i*_ along with *K*_*priv*_ forward the *PID*_*i*_ to KGC to generate partial private key *(Key*_*ppriv*_*)* to be used for further communication.
4. Using partial private key extraction algorithm, KGC generate *Key*_*ppriv*_ using conventional private key extraction algorithm, and return to *V*_*i*_.
5. Using *Key*_*ppriv*_ and *K*_*priv*_, *V*_*i*_ generate a new pair of public-private key to be used for subsequent communication among the entities. For this purpose, public-private key generation algorithm is used.
6. Using message *(m)*, *PID*_*i*_, and *K*_*priv*_, *V*_*i*_ generates a signature message, and forward to other concerning entities including RSU and vehicles.
7. Before authentication, if reconstruction is proceeded successfully, further communication is carried on; otherwise aborted immediately.
8. On other side, RSU or any other vehicle (*V*_*j*_) ensure the signature validation based on input parameters such as *m*, *PID*_*i*_, and *K*_*pub*_. *V*_*j*_ accepts the signature if valid otherwise reject and report accordingly.
9. On other hand, *V*_*i*_ may post a request for any point of interest (POI) to location service provider (LSP). In our mechanism, to reduce the communication overhead, we use cache based LSP that responds promptly. Otherwise, LSP post an external route request to online route API, and return the retrieved results back to requesting vehicle.

In this research, we use variety of algorithms including Pseudonym identity generation, Traceability, Partial-Private-Key-Extraction, Signature generation, and implementation of Lightweight Consortium Blockchain for RSUs in VANETs. Further detail of each algorithm is presented in algorithm 1,2,3,4, and 5 as follows.

**Algorithm 1.** Pseudonym identity generation.

**Inputs:**

 *RID*: **Real Identity of requesting vehicle** *Vi*

**Output:**

 *PID*: **Pseudonym Identity for requesting vehicle** *Vi*

**Declarations:**

 *Vi* → **Requesting vehicle**

**Process:**

 1. **if (***RID* **is valid) // Check validity of RID;**

 2. **then**

 3.        **generate** *PID***; // Generate a unique pseudonym identity;**

 4.        **store mapping (***RID*, *PID***) in the secure database;**

 5. **return** *PID* **to vehicle** *Vi*;

 6. **else**

 7.    **if (RID is invalid)**

 8.    **then**

 9.        **reject request and notify** *Vi*;

**Algorithm II.** Traceability of malicious vehicle.

**Inputs:**

 *PID*_*i*_: **Pseudonym Identity of suspected malicious** *Vi*

**Output:**

 *RID*_*i*_: **Real Identity of requesting vehicle Vi**

**Declarations:**

 *TA* → **tracing authority**

 *FI*→ **fake information**

 *McE*→ **Misconducting event**

 *L*_*msg*_→ **log of vehicles messages**

 *DB* → **Secure database**

**Process:**

 1. *TA* **Monitoring the VANET system.**

 2. **if** *(L*_*msg*_ *containing (McE or FI)*

 3. **Then**

 4.         **Trace** *PID*_*i*_ **of originating** *McE or FI*

 5.          **if (***PID*_*i*_ **found in** *DB***)**

 **6**.          **Then**

 7.                   **retrieve corresponding** *RID*_*i*_

 8.                   **broadcast** *RID*_*i*_ *& PID*_*i*_ **over network**

 9.                   **return** *PID*_*i*_ **for penalty imposition**

 **10.else**

 11.**return indicating unknown** *PID*_*i*_

**Algorithm 3.** Partial-Private-Key-Extract Algorithm.

**Inputs:**

 ***PID***_*i*_**: Pseudonym Identity for requesting vehicle *V***_*i*_

 **Key**_*priv*_**: Private key shared by V**_*i*_

**Output:**

 **Key**_*ppriv*_**: partial private key for subsequent V**_*i*_

 **Key**_*ppub*_**: partial public key for subsequent V**_*i*_

**Declarations:**

 ***S***_*i*_ → **random secret key**

 ***P*** → **generator point on the elliptic curve**

**Process:**

 1. **Receive *PID***_*i*_ **​and *Key***_*priv*_ **from *V***_*i*_

 2. **if (PID**_*i*_**​ is valid) // Validate the pseudonym identity of subsequent vehicle​;**

 **3**. **then**

 4.        **generate a random secret *S***_*i*_**​;**

 5.        **calculate the partial private key *Key***_*ppriv*_ **​ = *S***_*i*_**​.**

 6. **calculate the partial public key *Key***_*ppub*_ **​ = *S***_*i*_**​.*P* where *P* is generator point on the elliptic curve.**

 7.        **store the mapping of *Key***_*ppriv*,_ **Key**_*ppub*, *and*_ **PID**_*i*_ **in secure DB**

 8.        **return *Key***_*ppriv*,_ **Key**_*ppub*,_ **to V**_*i*_

 **9**. **else**

 10.**return error message indicating invalid *PID***_*i*_**​;**

**Algorithm 4.** Signature generation mechanism.

**Inputs:**

 *m*: *message content*,

 *PID*_*i*_: **Pseudonym Identity of subsequent** *V*_*i*_

 *Key*_*priv*_: **Private key generated by** *V*_*i*_

**Output:**

 *σ***: Signature**

**Declarations:**

 *F*_*q*_**​** → **finite field**

 *P* → **generator point on the elliptic curve**

 *q* → **a large prime number**

 *R* → **elliptic curve point**

 *k* → **random nonce from** *0 to n-1*. **Where n is total number of vehicles**.

**Process:**

 1. **Receive** *m*, *PID*_*i*_, *and Key*_*priv*_ **from** *V*_*i*_.

 2. *k* **= Generate a random nonce** *k* **from the finite field** *F*_*q*_**​**

 3. **Calculate** *R* **=** *k*. *P*

 4. **If (R is not NULL or Empty)**

 5. **{**

 6.         **Calculate r = hash (***R***) mod** *q*

 7.         **Calculate s = (hash (m) + r.** *Key*_*priv*_**)**^*k-1*^ **mod** *q*

 8.  *σ* **= (r,s)**

 9. **}**

 10.**Else**

 11.**{**

 12.         **Terminate process with message “Invalid input value.”**

 13.**}**

 14.**return** *σ*

**Algorithm 5.** Lightweight Consortium Blockchain for RSUs in VANETs.

**Inputs:**

 *T*_*i*_: **Transaction from vehicle** *V*_*i*_,

 *PID*_*i*_: **Pseudonym Identity of subsequent** *V*_*i*_

 *σ***: Signature of the transaction**

**Output:**

 *LCBC***: lightweight consortium blockchain**

**Declarations:**

 *CBC***​** → **consortium blockchain**

 **H** → **Cryptographic hash function**

 *k* → **Nonce for proof-of-work**

 *M* → **Merkle root of transactions**

 *H*_*prv_block*_ → **hash of previous block.**

 *RSU*_*kpub*_ → **public key of RSU.**

**Process:**

 1. **Receive** *T*_*i*_, *PID*_*i*_, *and σ*_*i*_ **from** *V*_*i*_.

 2. **Verify** *σ*_*i*_ *using PID*_*i*_, *and RSU*_*kpub*_.

 3. **if (Verify (***σ*_*i*_ *by PID*_*i*_, *and RSU*_*kpub*_**) = true)**

 **4**. **then**

 5.         **append verified** *T*_*i*_ **to the pending transactions list.**

 6.         **construct a new block:**

 7.         **gather pending transactions and form a block data structure.**

 8.         **compute the** *M* **of the transactions.**

 **9**.         **compute the proof-of-work:**

 10.        **set** *k* **= 0**

 11.        **for** *H* **(***H*_*prv_block*_ **||** *M* **||** *k***) meets the difficulty target**

 12.                **increment k.**

 13.**Form the new block with** *k*, *M***, and the hash of the previous block.**

 **14.else**

 15.**Discard** *T*_*i*_**, and report.**

 16.**return** *σ*

## VI. Assumptions for proposed scheme

System assumptions play a crucial role in defining the operational context and expected behavior of a VANET architecture. Here are some typical system assumptions for the described VANET architecture:

1. ***Secure Channels*:** All communication channels between Vehicles, OBUs, RSUs, TA, KGC, LSP, and the consortium blockchain network are assumed to be secure and resistant to eavesdropping or tampering.
2. ***Correct Operation of Cryptographic Algorithms*:** Cryptographic algorithms, including encryption, digital signatures, and hash functions, operate correctly and are resistant to known attacks.
3. ***Consensus Algorithm Reliability*:** The consensus algorithm used in the blockchain network is reliable and ensures the integrity of the distributed ledger. The assumptions include the correctness of the consensus mechanism, resistance to attacks, and the absence of malicious actors with significant control over the network.

These assumptions collectively set the context for the VANET architecture’s expected operational environment and guide the design and analysis of the system. It’s essential to review and validate these assumptions periodically to ensure their continued relevance as the VANET ecosystem evolves.

## VII. Security analysis

This section illustrates that the proposed scheme effectively fulfills the security and privacy criteria outlined for vehicular communication in the design objectives subsection. Following are possible attacks and their resistance using our proposed scheme.

### A. Query content modification attack resistance

Let *S* represent the set of legal vehicle-related sensitive information, and let ′*S*′ represent the set of pseudonym data forwarded by the anonymizer server. Additionally, let *C* represent the cloaking region generated by the anonymizer server and forwarded to the RSU. Then, the lemma can be expressed as follows:

**Lemma.** For any adversary *A* attempting to tamper with the legal vehicle-related sensitive information *S*, such that *S*≠*S*′, in the presence of the anonymizer server and the proposed scheme, the adversary’s ability to modify *S* is significantly impeded. Mathematically, this can be represented as in Eq 1.


$$\forall S\in S,\forall A:P(A\;modifies\;S|S\neq S\prime ,C)\ll P(A\;modifies\;S|S=S^{\prime },C)$$ (1)

Where *P* (*A* modifies *S* ∣ *S*≠ *S*′, *C*) denotes the probability of the adversary *A* successfully modifying the legal vehicle-related sensitive information *S* when *S* is different from the pseudonym data ′*S*′ and the cloaking region *C* is present. *P* (*A* modifies *S* ∣ *S* = *S*′, *C*) denotes the probability of the adversary *A* successfully modifying the legal vehicle-related sensitive information *S* when *S* matches the pseudonym data ′*S*′ and the cloaking region *C* is present.

### B. Content replay attack resistance

For any attacker *A* attempting to perform a replay attack by using a different timestamp ′*T*′ in place of the original timestamp *T*, the proposed system effectively detects and rejects the replayed message. Eq 2 presents it mathematical form:

∀T,T′∈Timestamps,∀A:P(AT′)≪P(AT) (2)

Where *P* (*A* successfully replays message with *T*′) denotes the probability of the attacker *A* successfully replaying a message with a different timestamp ′*T*′. *P* (*A* successfully replays message with *T*) denotes the probability of the attacker *A* successfully replaying a message with the original timestamp *T*. This proof indicates that the proposed system effectively mitigates replay attacks by verifying the freshness of timestamps in traffic-related messages. Since using a different timestamp results in a different value for the inequality check, replayed messages are promptly detected and rejected.

### C. Impersonation attack resistance

Let The combination of consortium blockchain, Certificateless Aggregate Signature (CLAS), and conditional privacy-preserving mechanisms provides resistance against impersonation attacks in VANET systems. The lemma against impersonation attack resistance can be formulated as follows:

**Lemma.** In a VANET system, each vehicle *V*_*i*_​ communicates securely by generating a pseudonym identity *PID*_*i*_​ and a private key *K*_*priv*_, which are used in conjunction with a consortium blockchain and CLAS. When *V*_*i*_ ​sends a message mim_imi​, it generates a signature *σ*_*i*_ using *K*_*priv*_ ​and aggregates it with signatures from other vehicles to form an aggregate signature *σ*_*i*_​. The aggregated signature *σ*_*iagg*_ = $\prod_{i=1}^{n}\sigma i$ is verified using an aggregated public key *PK*_*agg*_​, ensuring the authenticity of all *n* messages *m*_*i*_​ without relying on individual certificates. The consortium blockchain ensures data integrity and traceability by maintaining a distributed ledger, where each block contains a hashed record of transactions *T*_*i*_​. Conditional privacy-preserving mechanisms allow trusted authorities to map pseudonym identities *PID*_*i*_​ back to real identities *RID*_*i*_​ ​only under specific conditions, preventing unauthorized tracking. If an adversary *A* attempts an impersonation attack by forging a pseudonym *PID*_*A*_​ ​and a corresponding signature *σ*_*A*_​, the absence of a valid private key *K*_*privA*_ linked to *PID*_*A*_ ​ means *σ*_*A*_ ​ will fail verification against *PK*_*agg*_​. The integrity checks and traceability offered by the consortium blockchain further thwart the attack by detecting inconsistencies. Hence, the integration of consortium blockchain, CLAS, and conditional privacy mechanisms effectively resists impersonation attacks by ensuring only valid, authenticated communications are accepted in the VANET system.

Eq 3 express the formal representation as follows:


Verify(PKagg​⋅PKA​,σagg​⋅σA​,m1​,m2​,…,mn​,mA​)=False (3)

### D. Middle-man attack resistance

Let *S* denote the sender of a message, *V* denote the verifier, and *M* represent the message transmitted in the VANET system. The lemma for Resistance to Man-in-the-Middle (MITM) Attack in the described scenario can be formulated as follows:

**Lemma.** In the VANET system architecture described, incorporating an anonymizer server connected with the On-Board Unit (OBU), tracing Authority (*TA*) within the core network containing the cache based Location Based Service Provider (LSP) followed by linkage with blockchain and further blockchain network, the system effectively resists Man-in-the-Middle (MITM) attacks. Mathematically, this can be expressed as in Eq 4:

∀M,S,V:P(SVrelationshipisverified)=1 (4)

Where *P* (*SV* relationship is verified) denotes the probability that the relationship between the sender *S* and the verifier *V* is verified, ensuring that the message *M* is transmitted securely without intervention. *M* represents the message transmitted in the VANET system. It shows that in the described system architecture, the verification of the relationship between the sender and the verifier is a necessary condition for ensuring message validity and authenticity. By establishing this relationship and ensuring that a genuine message cannot be changed or fabricated, the system effectively mitigates the risk of Man-in-the-Middle (*MITM*) attacks.

### E. Trade-off analysis

Analyzing the proposed consortium Blockchain and certificateless conditional Privacy Protection (CPP) scheme for VANET systems, we must consider the trade-offs between security, privacy, and computational efficiency. Let *S*, *P*, and *E* represent security, privacy, and efficiency, respectively. The proposed scheme leverages the decentralized and immutable properties of blockchain technology to enhance *P* by ensuring that no single entity has undue control over sensitive information, thus reducing the risk of privacy breaches (*P↑*). The use of a decentralized identity management system, *TA*, *CLAS*, *CPP*, and consensus mechanisms provides a robust framework for transaction validation and authorization within the VANET, enhancing *S* by mitigating the risk of malicious attacks (*S↑*).

However, the introduction of these components can potentially increase the computational overhead (*E↓*), as blockchain operations inherently involve complex cryptographic processes and consensus mechanisms. The evaluation of the proposed scheme, which demonstrates improved privacy and efficiency, suggests a careful balance between these factors. The performance metrics, including reduced computation time (*E↑*), communication overhead (*E↑*), and improved precision, recall, F-measure, and ADP, indicate that the scheme is optimized to enhance *E* while maintaining high levels of *S* and *P*. Thus, the trade-off analysis reveals that the proposed scheme successfully enhances *P* and *S* without significantly compromising *E*, making it a viable solution for the privacy and security challenges in VANET systems.

## VIII. Experiments and results

Our simulation framework incorporates multiple components including Veins [56] which is basically combination of two simulation tools such as OMNeT++ [57], and SUMO [58]. To assess the performance of the proposed privacy provisioning model, we utilize established metrics such as precision, recall, F-measure, false-positive rate, and EDP (Expected Detection Probability).

Furthermore, we illustrate computational and communication cost of our model, and compare with existing state-of-the-art methods proposed in [37–39]. In the simulated road network, we deploy 200 vehicles accompanied by TA, KGC, LSP, 10 RSUs randomly positioned across the map which are linked through consortium blockchain.

At the initiation of the experiment, each vehicle is assigned a trust value of 0.2, acknowledging the time required for trust establishment in the system. Commencing with a lower trust value, like 0.1, would protract the differentiation between honest and malicious vehicles, while starting with a higher value, such as 0.6, would extend the time to reduce the trust value of a malicious vehicle, impeding detection. Therefore, a commencement value of 0.3 is considered apt. The communication range of each vehicle is set at 300m, with speeds ranging from 20 to 30m per second, randomly changing directions at intersections without pauses. Moreover, a road event is generated randomly within the network, and neighbouring vehicles witness the event, sending anonymized messages to the closest RSU within their communication range. Nevertheless, malicious vehicles attempt to sabotage the network by transmitting false messages to the nearby RSU, falsely refuting any occurrence of an accident. The majority of vehicles in the network exhibit legitimate behaviour, with the count of legitimate vehicles held constant. The trust model’s response is evaluated with the introduction of malicious vehicles, varying their percentage from 10% to 50%. This variation aims to confirm the effectiveness of our proposed scheme in detecting malicious vehicles and their deceptive data. The RSU calculates the trust value for each involved vehicle, verifies the legitimacy of the event, broadcasts a notification to vehicles within its communication range, and propagates a block containing the trust values of the vehicles across the blockchain network. Each simulation scenario is subjected to 10 runs with unique random seeds, guaranteeing varied initial node locations for each iteration. The detailed simulation parameters are presented in Table 3, providing comprehensive insights.

**Table 3:** Simulation attributes.

Attributes	Values
Simulation area	4 x 4 (KM)
Time	300 Seconds
Tracing Trusted authority	1
Key generation center	1
Communication range	300 m
Number of RSU	10
MAC Protocol	IEEE 802.11p

We conduct experiments eight times to accurately assess performance of VANET system by calculating average values for system time, average distance, connectivity, and privacy leakage entropy. We also calculate precision, sensitivity, balanced evaluation, proportion of legitimate messages, and Attack detection probability in our proposed model. The details are given as follows:

### A. Computation cost

To evaluate our proposed privacy provisioning framework, we evaluate different metrics where computation cost is the first one that refers to the computational resources required to perform various operations such as encryption, decryption, hashing, verification, and other processing tasks. This cost is crucial to consider as it directly impacts the efficiency and performance of the system. Mathematically, the computation cost *C* is expressed in Eq 5 as the sum of the costs associated with individual operations:

$$C(comp)=\sum_{i=1}^{n}C_{i}$$ (5)

**Fig 3:**
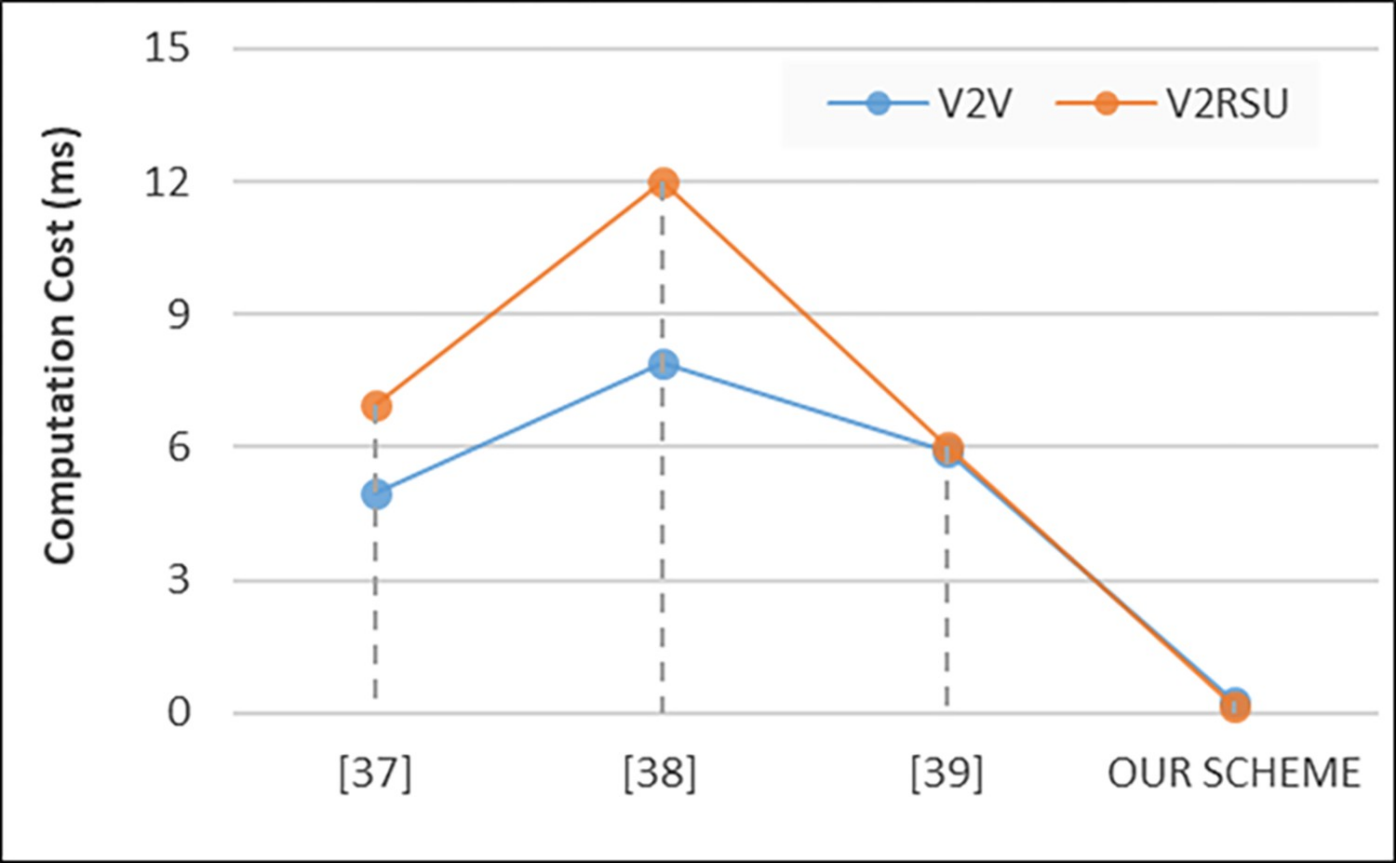
Computation cost for V2V and V2RSU.

Where, *C(comp)* is the total computation cost, *n* is the total number of operations, and *C*_*i*_​ represents the cost of each individual operation. The computation cost for different operations can vary depending on factors such as algorithm complexity, key length, message size, and processing power of the devices involved. To calculate the computation cost in VANET systems, one must consider the specific operations involved in tasks such as encryption for securing communications, hashing for data integrity verification, signature generation and verification for authentication, and any other cryptographic operations necessary for privacy protection and security. Additionally, factors such as the number of vehicles, the frequency of message exchanges, and the computational capabilities of the network nodes need to be taken into account for accurate computation cost estimation. The computation cost analysis is illustrated in Fig 3 as outlined below. In terms of V2V communication, our scheme demonstrates a significantly lower computational cost compared to the referenced schemes, with a value of 0.23972. This indicates a substantial reduction in computational overhead, enhancing efficiency. Similarly, for V2RSU communication, our scheme exhibits a substantially lower computational cost of 0.13166 compared to the referenced schemes, further emphasizing the efficiency gains achieved. These results suggest that our proposed scheme effectively minimizes computational costs, making it a promising solution for VANET systems.

### B. Communication cost

In our experiments, the second evaluation metric is communication cost that refers to the resources expended in transmitting data between vehicles, roadside units (RSUs), and other network components. It encompasses factors such as bandwidth usage, message overhead, and transmission delays. Mathematically, the communication cost *C(com)*​ is expressed in Eq 6 as follows:

$$C(com)=\sum_{i=1}^{n}(B_{i}\;X\;T_{i})$$ (6)

**Fig 4:**
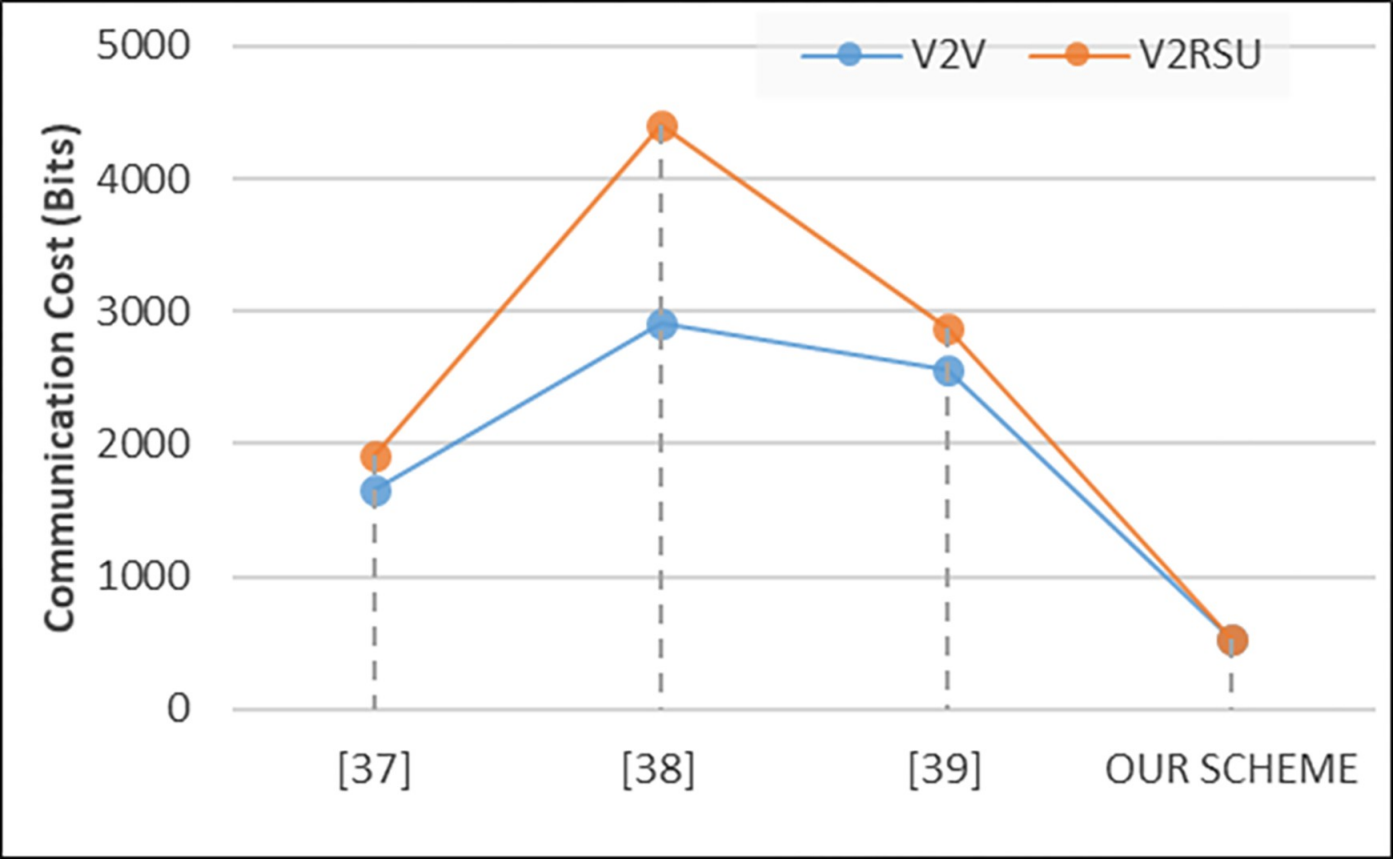
Communication cost.

Where, *Bi*​ represents the bandwidth consumption of the i^th^ communication link, *Ti*​ denotes the transmission time for data over the i^th^ link, and *n* is the total number of communication links in the network. In our experiments, analysis of communication cost evaluation across various schemes, including references [37–39], compared to our proposed scheme, reveals significant differences in resource utilization. According to Fig 4, in the case of V2V communication, our scheme demonstrates notably lower communication costs, with 534 and 530 bits compared to 1664, 2912, and 2560 bits for references [37–39], respectively. Similarly, for V2RSU communication, our scheme exhibits reduced communication costs, registering values of 530 compared to 1920, 4416, and 2880 in references [37–39], respectively. These findings underscore the efficiency and optimization achieved in communication resource utilization through our proposed scheme, suggesting its superiority in minimizing communication overhead in VANET systems.

### C. Cache hit ratio

We evaluate the effective usage of cache while receiving queries from the vehicles. In a VANET system employing a cache-based anonymizer server situated between OBUs and RSUs, the cache hit ratio *R*_hit_​ represents the proportion of queries successfully resolved from the cache compared to the total number of queries made. It can be expressed as in Eq 7:

$$R_{hit}=\frac{N_{hit}}{N_{query}}$$ (7)

**Fig 5:**
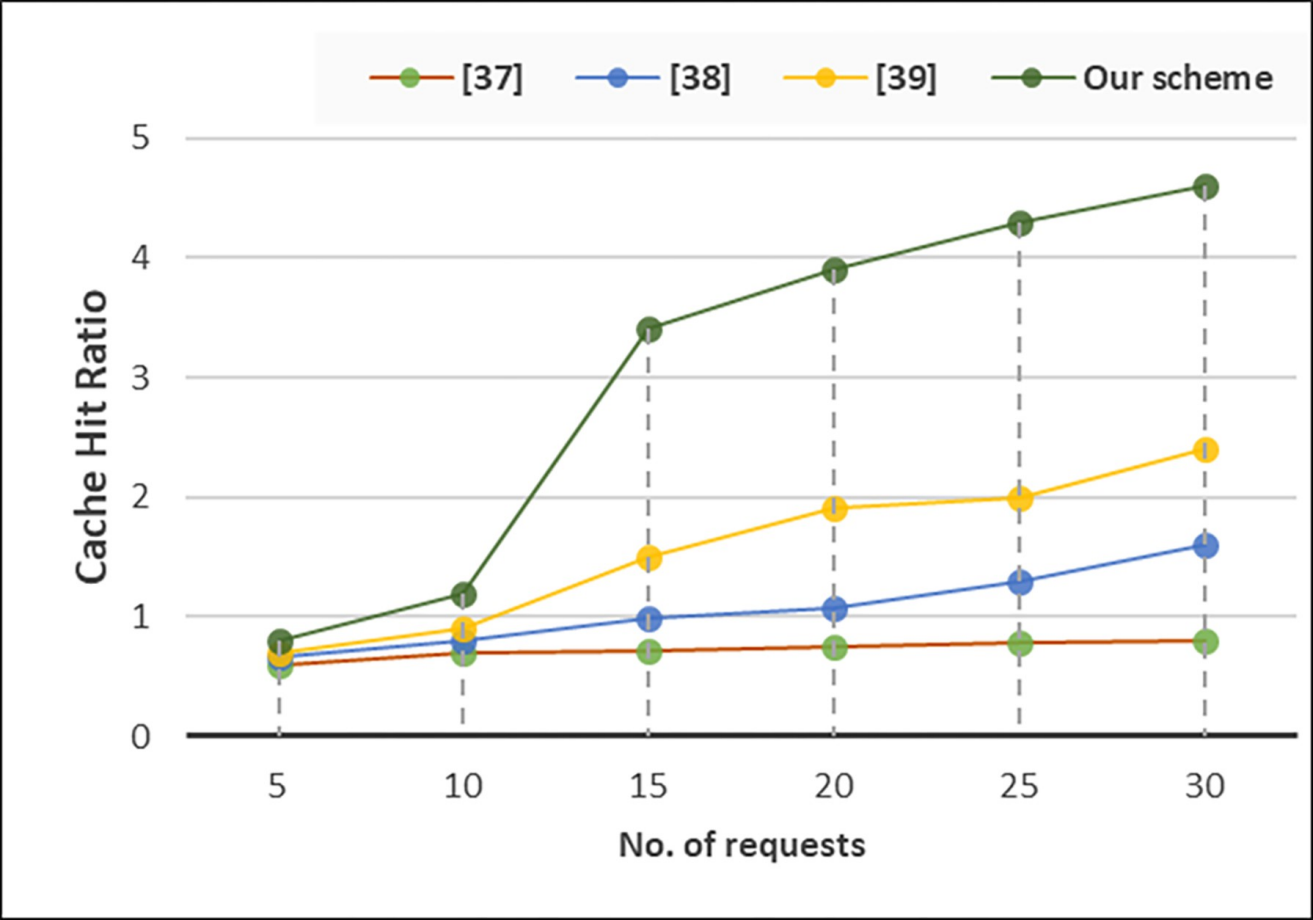
Cache hit ratio.

Where, *N*_*hit*_​ is the number of queries successfully resolved from the cache, and *N*_*query*_​ ​is the total number of queries made by OBUs to the anonymizer server. Fig 5 presents the impact of cache usage that significantly improve the overall system. We observe that at initial level, cache hit ratio is almost in all methods, but a significant difference once the number of queries increased.

### D. Location Leakage Probability (LLP)

To calculate the location leakage probability in VANET systems, we define it as the probability that an adversary can accurately infer the location of a vehicle based on the information available to them. LLP can express as in Eq 8 as follows.

$$P\;(Loc\;Leakage)=\frac{No.of\;successful\;loc\;interference}{Total\;no.of\;requests}$$ (8)

**Fig 6:**
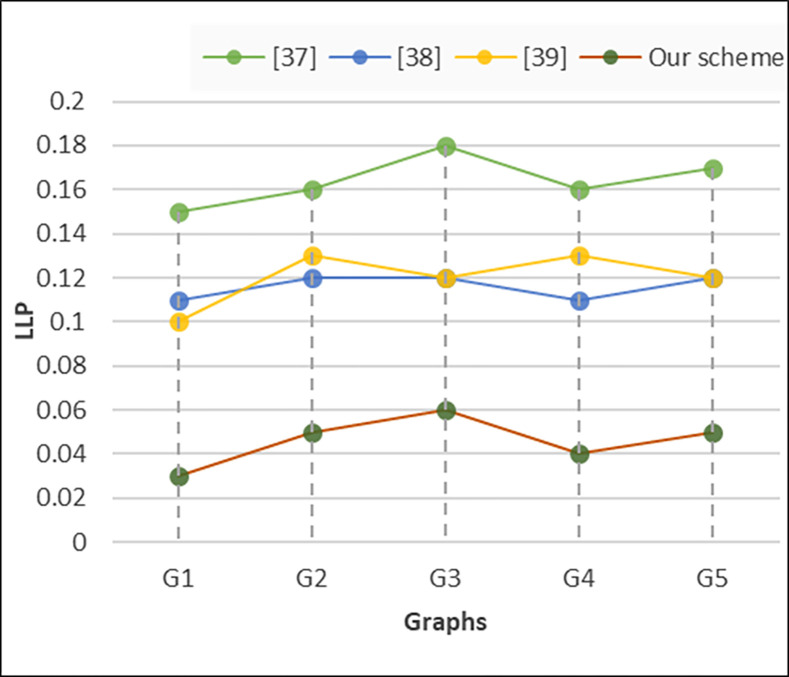
Location leakage probability.

Where *P* (Location Leakage) is the location leakage probability, "Number of Successful Location Inferences" represents the instances where the adversary correctly identifies the location of the vehicle, and "Total Number of Location Queries" denotes the total number of times the adversary attempts to infer the location of the vehicle. This evaluation can be performed over a given period or set of scenarios to assess the effectiveness of privacy protection mechanisms in the VANET system. As depicted in Fig 6, the probability LLP stands at 0.15 with centralized architecture, ranging between 0.1 and 0.12 with distributed architecture, and between 0.1 and 0.13 with cache-based centralized architecture. In contrast, our proposed architecture lowers the probability to approximately 0.05%. Consequently, the effectiveness of LLP protection is significantly enhanced with our proposed architecture.

### E. Precision

In VANET (Vehicular Ad-Hoc Network) systems, precision is a metric used to evaluate the accuracy of a system in correctly identifying relevant instances among the total instances that it has identified as positive. Precision is particularly relevant in the context of security and trust models, where it measures the proportion of correctly identified positive instances among the instances identified as positive. The precision is calculated using Eq (9) as follows:

$$Precision=\frac{True\;positive\;(TP)}{True\;positive\;(TP)+False\;positive\;(FP)}$$ (9)

**Fig 7:**
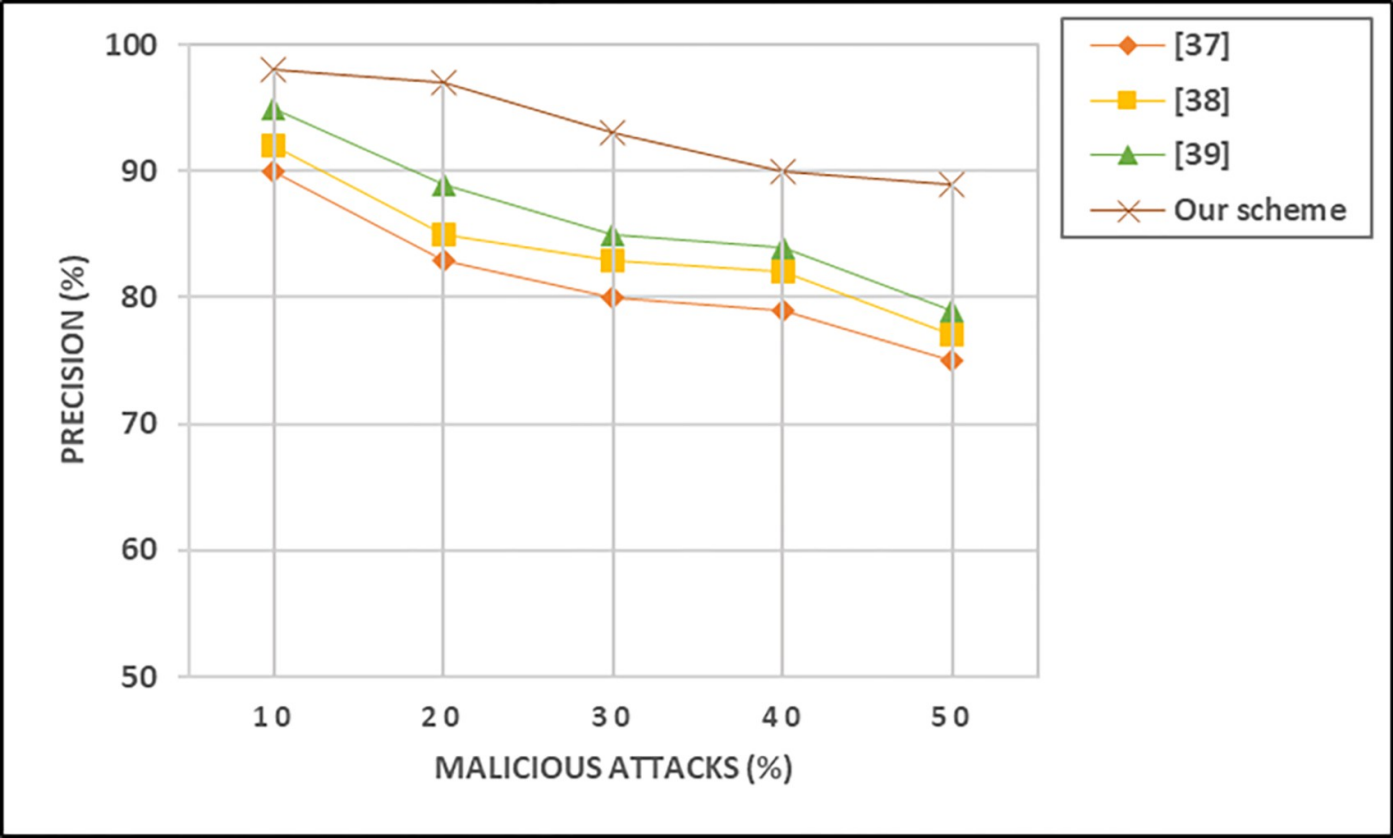
Influence of different proportions of malicious vehicles on precision.

In the context of VANET systems, precision can be applied to various aspects, such as the accuracy of identifying trustworthy vehicles or the precision of detecting security-related events. A higher precision value indicates a lower rate of false positives, signifying a more accurate positive identification by the system. The precision of the privacy provisioning models is depicted in Fig 7. It indicates that in defined scenarios with a limited number of malicious vehicles, all four trust models achieve a precision surpassing 90%. This highlights the proficiency of the trust models in accurately identifying malicious messages transmitted by these vehicles with malicious intent.

### F. Recall

Recall, also known as sensitivity or true positive rate, in VANET systems measures the ability of a trust model to correctly identify all relevant instances of malicious behavior, indicating the proportion of actual positive instances that were correctly detected. Eq 10 presents the mathematical form to calculate recall.


$$Recall=\frac{True\;positive\;(TP)}{True\;positive\;(TP)+False\;Nagative\;(FN)}$$ (10)

**Fig 8:**
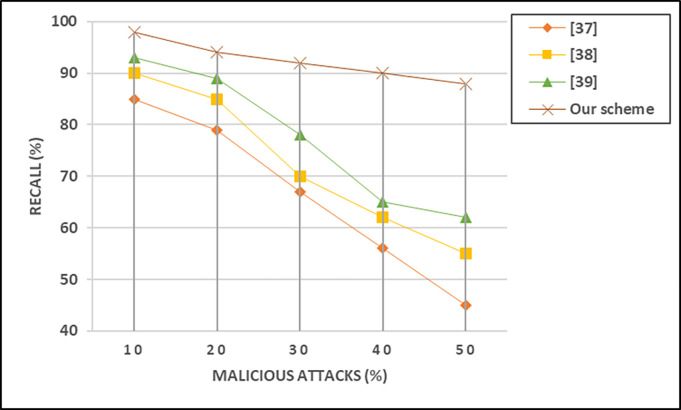
Recall impact while malicious attacks at different levels.

True Positive represents instances where the trust model correctly identifies malicious behavior, and False Negative represents instances where malicious behavior is present but the trust model fails to detect it. Our suggested scheme demonstrates superior performance compared to all existing methods in terms of recall, as illustrated in Fig 8. In situations where the network includes 50% malicious vehicles, our scheme achieves recall values that exceed the existing methods by 12%, 19%, and 22% respectively.

### G. F-measure (F1 score)

In VANET systems, the F-measure or F1 score is a metric that combines both precision and recall to provide a balanced evaluation of a system’s performance. It considers both the false positives and false negatives and is particularly useful when the classes are imbalanced. The F-measure is calculated using the following Eq 11 as follows:

$$F_{b}=\frac{{(1+b}^{2})*precision*recall}{b^{2}*precision+recall}$$ (11)

**Fig 9:**
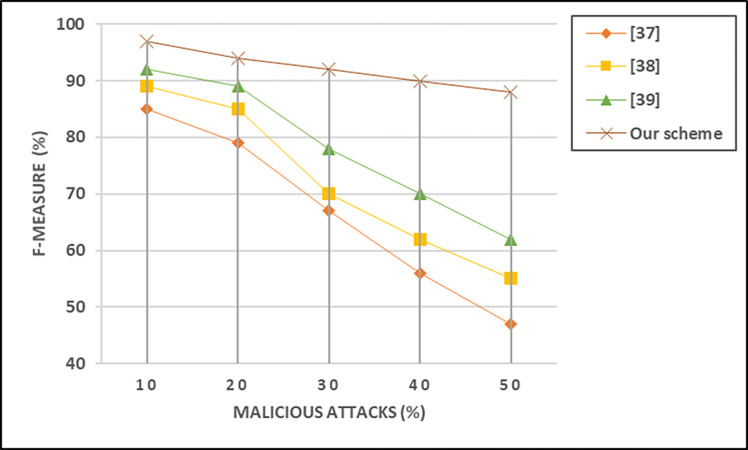
Impact of different malicious attacks on F-measure.

Fig 9 illustrate that our scheme outperformed the existing method, and achieved 18%, 22%, and 26% higher F-measures when the number of malicious attacks is at maximum range.

### H. False positive rate (FPR)

The false positive rate in VANET systems is a metric that quantifies the proportion of legitimate messages or events wrongly identified as malicious or unauthorized. In the context of VANET security, a false positive occurs when the system mistakenly flags a valid communication or activity as a security threat. The false positive rate is a crucial measure as it helps evaluate the accuracy of intrusion detection systems and other security mechanisms in distinguishing between normal and potentially harmful behavior. The formula to calculate the false positive rate is given as follows in Eq 12.


$$FPR=\frac{No.\;of\;False\;Positives}{No.\;of\;False\;Positives+No.\;of\;actaul\;negatives}$$ (12)

**Fig 10:**
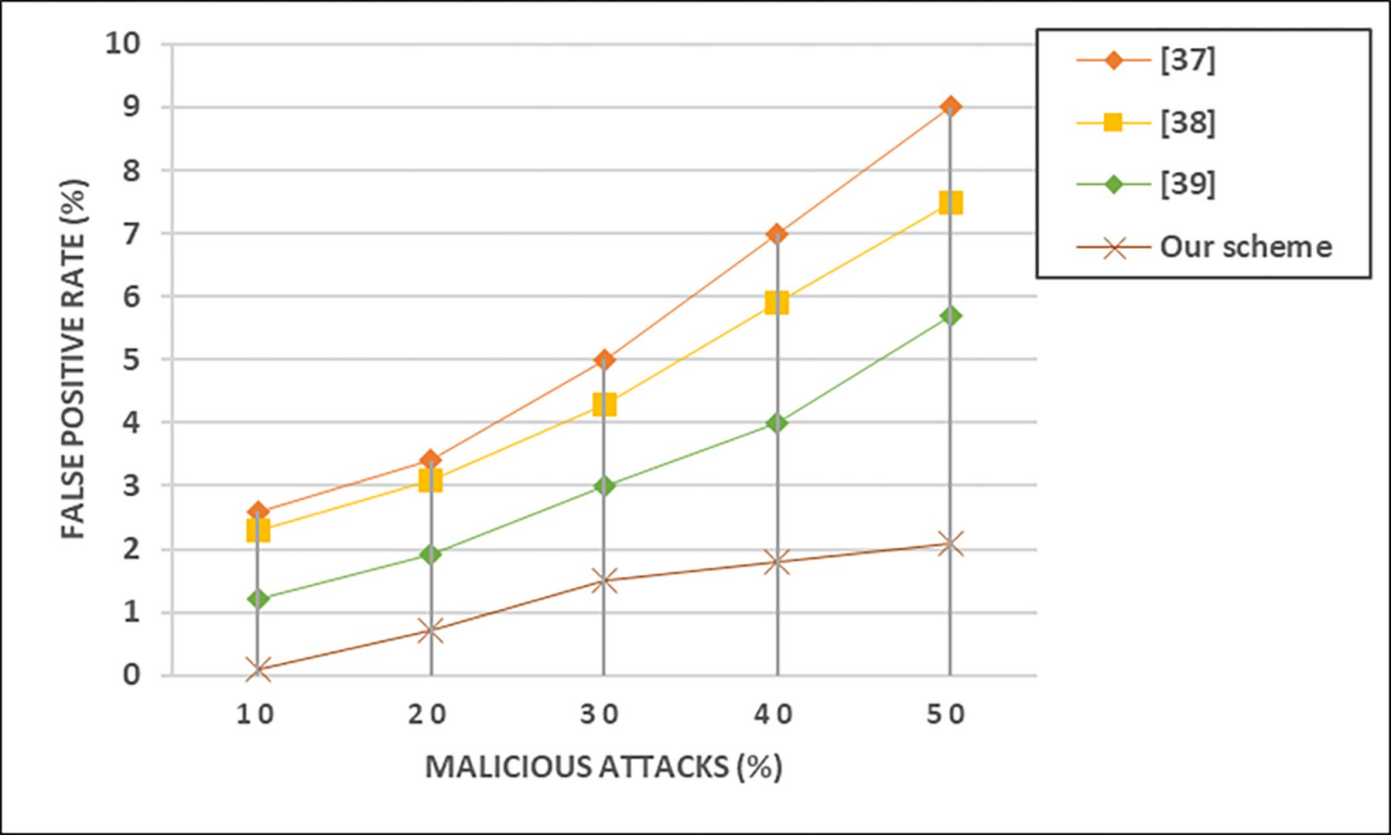
Impact of different malicious attacks on false positive rate (FPR).

This rate provides insights into the efficiency of the system in avoiding unnecessary alarms or alerts for non-malicious activities, contributing to the overall reliability of the security infrastructure in VANETs. Fig 10 shows the impact of different malicious attacks on false positive rate (FPR).

### I. Attack detection probability (ADP)

**Fig 11:**
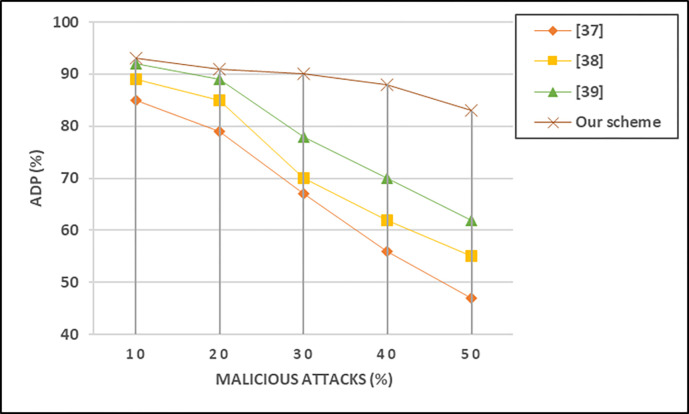
Probability of attack detection at different number of malicious attacks.

Attack detection probability in VANET systems is a metric that quantifies the likelihood of correctly identifying and detecting malicious activities or attacks within the network. It measures the effectiveness of the security mechanisms in place to recognize and respond to potential threats as shown in Fig 11. The attack detection probability is calculated using the following formula in Eq 13 as follows:

$$ADP=\frac{No.\;of\;detected\;attacks}{No.\;of\;actaul\;attacks}$$ (13)

We conducted a comprehensive assessment and comparison to gauge the effectiveness of the proposed authentication scheme and trust model in relation to other pertinent authentication schemes and trust models. The evaluation of the authentication scheme takes into account factors such as computation and communication overhead, along with accuracy. The performance of existing privacy provisioning models, including our proposed solution, is scrutinized based on metrics such as precision, recall, F-measure, False Positive Rate (FPR), and Attack Detection Precision (ADP). The results unequivocally demonstrate the superior performance of our proposed scheme, showcasing reduced computation time and communication overhead compared to alternative schemes. Furthermore, our scheme outperforms the compared models by achieving elevated precision, recall, F-measure, and ADP.

## IX. Conclusion

In the rapidly expanding realm of connected vehicular ad hoc networks (VANETs), the paramount concern is ensuring robust privacy and efficiency. This paper introduces a groundbreaking consortium Blockchain and certificateless conditional Privacy Protection scheme for VANET systems. By harnessing the decentralized and immutable attributes of blockchain technology, the proposed scheme establishes a robust framework for preserving sensitive information within VANETs. The key components, including a decentralized identity management system, Trusted Authority (TA), CLAS, CPP, and consensus mechanisms, collectively validate and authorize transactions within the VANET. The decentralized nature of the blockchain ensures that no single entity has undue control over sensitive information, mitigating the risk of privacy breaches. A comprehensive evaluation of the proposed scheme, encompassing data privacy, system performance, and resistance to malicious attacks, underscores its significant enhancement of VANET system privacy and efficiency. This research contributes to the evolving discourse on privacy protection and efficiency in connected vehicular environments, addressing challenges in the era of smart transportation. The extensive assessment and comparison of the proposed authentication scheme and trust model against relevant counterparts establish their superior performance in terms of reduced computation time, communication overhead, and enhanced precision, recall, F-measure, and ADP.

By future perspectives, our aim is to implement the proposed model in real world environment to observe its effectiveness. Furthermore, VANETs always refers to increase efficiency as much as we can. Therefore, we will add an additional layer called Fog computing layer with massive parallel computing approach to enhance efficiency of the system.
